# John Hunter and War Surgery

**Published:** 1917-02-24

**Authors:** 


### The Hospital

The Workers' Newspaper of Administrative Medicine and Institutional Life, Administration, National Insurance and Health.

No. 1603, Vol. LXI. SATURDAY, FEBRUARY 24, 1917. PRICE ONE PENNY.

### JOHN HUNTER AND WAR SURGERY.

The tradition of the annual Hunterian Oration at the Royal College of Surgeons is both a long and a dignified one, and it would truly have been a matter for regret had the emergency of the war caused it to be interrupted. The series commenced in 1814, and it has an unbroken record to the present date. If on individual occasions the story has been a twice-told tale, it may be remembered that the audience each year is new, or at least partially new, and that to praise famous men, even in non-original terms, is a worthy offering to a rising generation. Here, indeed, is one of the justifications of these annual memorials. They recall to us not merely the results which have been attained by the men whose names they enshrine, but also, and much more important, the personal qualities by which the attainment of these results was effected. In this respect there is a specially sympathetic note in the closing passages of the Oration delivered before the College by Sir George H. Makins on the 14th inst. Here in a few happy phrases Hunter is presented as struggling through long years against obstacles and difficulties, by no means free from critics and even from enemies, and yet by intrepidity of mind and vigour of application winning through to a secure position among the immortals. The hero is seen as no remote, shadowy figure detached from the conditions which govern ordinary human effort. On the contrary, the man and the soul that was within him make an appeal which comes home to the reader because of their essentially human qualities. It is less the genius of Hunter than his manhood which stirs the heart, and in emphasising this aspect of his subject the Orator of 1917 has been happily inspired. He has thought generously and sympathetically, and has spoken his thought in simple and effective phrases. Amidst the pressing and vital questions which survive from the past, as well as while contemplating those emerging from the new conditions of to-day, it is well for workers to hear that, whatever be their intellectual qualities and whatever their resources of equipment, they, not less than the giants of the past, must not fail to cultivate such homely virtues as industry, intrepidity, and vigour of application. The lesson may seem a trite one, but its repetition is ever necessary.

The main theme of the Oration also has a personal motive, and one which links Hunter to the surgeon of the day and hour. Its purpose is to set out the influence which military surgery exercises on the military surgeon. No one has a better right to speak on such a topic than Sir George Makins, and his sketch has the sure hand of the master. What Hunter experienced in the latter part of the eighteenth century Sir George and his colleagues are experiencing in the earlier years of the twentieth, and it is an interesting study to ask how in each case the work affected the man. Probably it will come as a surprise to many to read that essentially the difference between the two eras in regard to surgical practice is not a very wide one. The present war has revived proceedings which a time of peace had come to regard as definitely upon the scrap-heap, and as this is true of military methods, so also is it true of the practice of surgery. Old conditions have been re-established, and the means of meeting these to-day are found in principle to be those adopted by an older generation. "Some of the most modern surgical practice has been spoken of as a *Revolution*, yet the general tendency," says Sir George Makins, "has been rather in the direction of a *Reaction*." This is an interesting summing-up of the situation, and it by no means excludes a full recognition of improvement in details and methods. It is associated, too, with complete confidence in the sterling quality of the newer forms of laboratory work, which have taken on such an extensive development, not only in the large hospitals at the base but also well forwards towards the fighting-line. We quite agree with the tribute paid by the Orator to these enterprising workers, as also with the expectation that the knowledge thus acquired will have fields of application long after the present war has reached its appointed end. But for the practical surgeon at the bedside and in the operating theatre there is necessary, above all things, a firm hold of sound surgical principles, and this truth is one of those which experience of military work has impressed on the surgeon. It was taught to John Hunter by his experience and it is repeated to-day to his successors. With the illustrations of this truth depicted by Sir George Makins we have not here space to deal, but we are sure they will be recognised as both apt and forcible. We can refer only to one subject—namely, that of the local treatment of wounds, and as this has been the source of much controversy between champions of renown, it has a special interest at the moment, and a surgeon with large experience at the Front could hardly pass it over. Evidently, as was indeed inevitable, Sir George Makins has given the question much thought and has put the issue to the test over a wide area. His analysis of the controversy is both full and exact, and he has no mere forensic arguments or philosophical subtleties to urge. He makes a calm and reasoned contribution to a debate which is of high importance, and his verdict on the so-called "phylacogogic" method is not a favourable one. No surgeon should neglect this very significant part of the Oration.

Once more, on the personal aspect of military surgery, we learn that its practitioner between periods of active and unceasing work has "intervals of quietude which are rare in any other path of professional life." Thus he has time for thought and for following out lines of investigation and research. Out of these opportunities Hunter made many valuable observations and conclusions, and it cannot be doubted that some of our present military surgeons are following his example. There have already been substantial evidences that this is the case, and not the least convincing of these is the Oration which is our present text.

